# Association between Shift Work Schedules and Cardiovascular Events in a Multi-Ethnic Cohort

**DOI:** 10.3390/ijerph20032047

**Published:** 2023-01-22

**Authors:** Andrew Roshan Dicom, Xiangyuan Huang, Saima Hilal

**Affiliations:** Saw Swee Hock School of Public Health, National University of Singapore and National University Health System, Singapore 117549, Singapore

**Keywords:** shift work, cardiovascular disease, cardiometabolic disease, risk factors

## Abstract

Background: Shift work is known to increase the risk of cardiometabolic diseases and mortality. We investigate the relationship between shift work schedules and cardiometabolic risk factors (smoking, hypertension, and obesity) and their association with cardiometabolic diseases (diabetes and cardiovascular diseases) in a multi-ethnic population from Singapore. Methods: 2469 participants from the Singapore-based Multi-Ethnic Cohort underwent physical and clinical assessments. Shift work schedules (morning, evening, night, and mixed) were assessed using a validated questionnaire. Results: Among shift workers, night shift workers had a significantly higher prevalence of smoking (54.5%), diabetes (27.3%), and cardiovascular events (14.1%). Compared to non-shift workers, workers in the night (OR = 2.10, 95%CI: 1.26–3.41) and mixed (OR = 1.74, 95%CI: 1.22–2.48) shift groups were more likely to be current smokers. A significant association between shift duration and smoking (OR = 1.02, 95%CI: 1.00–1.03) was also observed, with longer shift duration (in years) leading to an increase in smoking behavior. No significant associations were found between shift work schedules and hypertension, obesity (BMI), diabetes, and cardiovascular disease, as well as other cardiometabolic risk factors and diseases. Conclusion: This study found that shift schedules and shift duration were most strongly associated with smoking status after covariate adjustments (age, gender, ethnicity, socioeconomic status, and work arrangement), with night and mixed shift types being strongly associated with current smoker status. As smoking is a modifiable risk factor for cardiometabolic disease, employers of shift workers should increase work-based health interventions to control smoking and promote a healthier workforce.

## 1. Introduction

Cardiometabolic diseases (CMD) including diabetes and cardiovascular disease (CVD) are a significant cause of morbidity, mortality, and burden of illness worldwide [1,2]. From 1990 to 2017, global prevalence rates, incidence rates, mortality, and disability-adjusted life years (DALYs) for diabetes significantly rose [3]. It is projected that the prevalence of diabetes will increase by 50% by 2050 [4]. Moreover, from 1990 to 2019, the prevalence rates of CVD doubled from 260 million to about 520 million, as global trends for DALYs and the number of years of life lost for CVD increased twofold in the same period [2]. Although mortality rates for CVD in developed countries have been declining in recent years, Singapore still has one of the highest mortality rates for CVD, such as coronary heart disease [1,5]. Similarly, data from the Singapore Stroke Registry Annual Report showed that between 2010 and 2019, the number of stroke episodes increased by 33% [6]. These trends indicate CMD to be a public health threat that is a major contributing factor to Singapore’s disease burden.

Shift work, defined as an employment period outside usual working hours (i.e., 7 a.m. to 6 p.m.) [7], is associated with an increase in adverse health outcomes, including CMD and mortality [8]. Recent studies have provided evidence for “a strong dose-response relationship” between shift work and the risk of CMD [9,10]. It has also been reported that shift workers are often at a higher risk of leading unhealthy lifestyles, such as having poorer dietary habits, lower physical activity, increased smoking, and poorer sleep quality than non-shift workers [11]. Although a direct causal pathway between shift work and chronic diseases has yet to be clearly delineated, there are numerous candidate mechanisms that link shift work to CMD [12]. These pathways include the behavioral or physiological reactions to shift work, including smoking, hypertension, and obesity [12]. Hence, there have been increasing calls for further research into this area [7].

To date, only one study in Singapore has directly investigated the possibility of negative health outcomes of shift work in a population-based setting, linking shift work with poor sleep quality [13]. Furthermore, very few studies have examined the relationship of shift work with cardiometabolic risk factors alongside cardiometabolic disease outcomes [12].

We investigate the relationship between shift work schedules (morning, evening, night, and mixed schedules) and cardiometabolic risk factors (smoking, hypertension, and obesity) and their association with cardiometabolic diseases (diabetes and CVD) in a multi-ethnic population from Singapore.

## 2. Methods

### 2.1. Study Population and Design

This study uses a cross-sectional design. The study population was taken from the Singapore Multi-Ethnic Cohort (MEC), which aims to study the determinants and risk factors of various chronic health conditions in three main ethnic groups in Singapore [14]. At baseline (phase 1), the MEC consisted of 14,729 participants. Recruitment was carried out between 2004 and 2010 via outreach and referrals. Participants recruited were Singapore citizens and long-term residents aged between 21–75 years old. In this initial phase, individuals with pre-existing non-communicable diseases (i.e., history of heart disease, stroke, etc.) were excluded.

The present study utilized existing data from the MEC cohort’s follow-up phase (revisit of phase 1) to assess the associations between shift work and health outcomes. In the revisit of phase 1, 6148 participants were followed-up between 2011 and 2016. Out of these, data from 6101 participants who had completed the interviews and questionnaires were available. Participants who did not provide or had missing shift work data were excluded from the analyses. Participants were also excluded if they had extreme values for their monthly shift durations (i.e., more than 30 days) and missing covariate data, such as age, gender, and socioeconomic status (SES). The remaining 2469 participants’ data were used for analysis (Supplementary Figure S1). 

All participants were contacted and visited at their homes or a convenient place of their choice for a structured interview. Health and lifestyle information (i.e., smoking, physical activity, medication, etc.) were collected during the interview. Participants also underwent a health screening at designated health screening sites following the interview. During the screening, anthropometric data (i.e., height and weight) and biosamples (i.e., blood and urine) were collected for analysis. All interviews and health screenings were conducted by trained study personnel to ensure consistency in the collection of data [14].

Ethics approval was provided by the National University of Singapore Institutional Review Board (IRB) (reference number: 12–140), and written informed consent was obtained before recruitment into the study [14].

### 2.2. Assessment of Shift Work

Shift work was assessed under the occupational physical activity section of a locally validated questionnaire, the Singapore Prospective Study Program Physical Activity Questionnaire (SP2PAQ) [15]. Firstly, participants were asked to provide the number of days per month they had to work in the evening, at night, or early morning. Then, participants were also asked to report on the number of years they had engaged in shift work in the evening, night, or early morning since they began working.

Shift schedules type: Shift types were derived by obtaining a weighted average of days per month (for a 30-day month) that participants had engaged in shift work. Shift work types consisted of four categories: evening, early morning, night, and mixed shift schedules. The evening shift was defined as any work occurring between 4 p.m. and 9 p.m., the night shift as being any work occurring between 9 p.m. and 6 a.m., the early morning shift as any work occurring between 4 a.m. and 8 a.m., and the mixed shift was a combination of two or more reported shift types occurring within a single month (i.e., evening and early morning shift). Participants were added to shift type categories if they had a value of 1 or more days spent in any shift work category _ENREF_16. Finally, workers who had a null value in all shift work categories were categorized as non-shift workers. 

Shift duration: Shift duration was derived from the weighted average of reported years engaged in shift work for evening, night, and early morning schedules, and was treated as a continuous variable.

### 2.3. Assessment of Cardiometabolic Risk Factors

Self-reported information on smoking status, hypertension, and anthropometric data were obtained through structured questionnaires and clinical assessments [14]. Smoking status was initially treated as binary data, whereby participants were grouped into ever smoker and never smoker. We further categorized ever smokers into past and current smokers. Body mass index (BMI) was derived from height and weight. BMI was treated as a binary variable and categorized into <25 kg/m^2^ (non-overweight) and ≥25 kg/m^2^ (overweight) [16]. Similarly, hypertension was also treated as a binary variable and categorized into a self-reported status of yes or no.

### 2.4. Assessment of Cardiometabolic Disease 

Diabetes was assessed via both interviewer-administered questionnaires and during the clinical assessment. Medication use and laboratory results for fasting blood glucose were used to confirm self-reported diabetes. Participants who reported medication use and/or fasting blood glucose results of ≥7.0 mmol/L were confirmed as being diabetic. Diabetes was treated as a binary variable and categorized as yes or no.

Cardiovascular disease (CVD) was defined as any of the following conditions: a blockage in any arteries, stroke, transient ischemic attack (TIA), heart failure, heart attack, and irregular heartbeat. CVD was assessed via both interviewer-administered questionnaires and during the clinical assessment. Information on medication intake or medical procedures (i.e., heart bypass operation, usage of a pacemaker, angioplasty-ballooning, etc.) was also used to identify participants with cardiovascular disease. CVD was treated as a binary variable and categorized as yes or no.

### 2.5. Assessment of Other Covariates

Data such as age, gender, ethnicity, and work arrangement were assessed via interviewer-administered questionnaires. Age was treated as a categorical variable and categorized into four age bins: <35, 35–44, 45–54, and ≥55. Gender was categorized into male and female. Ethnicity was categorized into four sub-categories: Chinese, Malay, Indian, and others. Work arrangement (i.e., part-time or full-time work) was calculated by multiplying the self-reported number of hours worked per day by the self-reported number of days worked per week. Participants who worked less than 35 h a week were listed as part-time and those who worked 35 h or more per week were listed as full-time, based on criteria by Singapore’s Ministry of Manpower (MOM) [17]. 

Socioeconomic information such as household income, housing type, and education level were assessed via interviewer-administered questionnaires. Low SES was derived from household income, housing type, and education level. Participants were placed in the low SES grouping if they met the following conditions: household income equal to or less than 2000 (SGD), having no or low (below A-level) education, and living in a four-room housing development board flat or smaller. Low SES was treated as a binary variable and categorized as yes or no.

### 2.6. Statistical Analyses

All analyses were performed using IBM SPSS Statistics for Macintosh (IBM SPSS Statistics for Macintosh, Version 27.0. Armonk, NY, USA: IBM Corp) [18].

Descriptive data were compared using Pearson’s chi-square test and one-way analysis of variance (ANOVA). Categorical data such as gender, age range, ethnicity, low SES, smoking status, hypertension occurrence, BMI, diabetes, and CVD were compared with shift-type using Pearson’s chi-square test for independence. A one-way ANOVA was used to compare shift schedule types (categorical) and shift duration (continuous) with the outcome of interest.

Binary and multinomial logistic regression models were used to examine associations between shift type and shift duration (in years) with CMD risk factors (smoking status, hypertension, and BMI) and CMD (diabetes and CVD) with the relevant odds ratio (OR) and 95%CI. All analyses were adjusted for gender, age range, ethnicity, and low SES as potential confounders [19,20]. We created the models in the following fashion: in the crude model, no covariates were added in the model; Model 1 was adjusted for sociodemographic factors of age, gender, ethnicity, and SES; Model 2 was adjusted for model 1 and work arrangement.

## 3. Results

### 3.1. Characteristics of the Study Population

Out of 6148 participants, 2469 provided shift work information and were included in the final analyses, of which 638 (26%) were shift workers, and 1831 (74%) were non-shift workers. Of the 638 shift workers, 47 (7.4%) were on the early morning shift, 268 (42.0%) were on the evening shift, 99 (15.5%) were on the night shift, and 224 (35.1%) were on mixed shift schedules.

### 3.2. Differences between Shift Schedule Types

Table 1 shows the demographic characteristics of shift workers, stratified according to their shift schedules (morning, evening, night, and mixed). A significant difference was observed in the length of years spent doing shift work between shift workers (*p* = 0.027). On average, workers on night shift schedules spent the greatest number of years doing shift work, while those in the evening shift schedules spent the least number of years in shift work. Chi-square analyses showed significant associations for shift type with work arrangement (*p* = 0.001), gender (*p* < 0.001), age range (*p* < 0.01), ethnicity (*p* < 0.001), low-SES (*p* < 0.02), smoking status (*p* < 0.001), diabetes (*p* < 0.01), and CVE (*p* < 0.01). Overall, less than 20% of workers in all shift types worked in part-time arrangements (less than 35 h a week), except for evening shift workers (20.9%). Night (67.7%) and mixed (67.9%) schedules had a higher proportion of male than female workers. Night shift workers also tended to be older, with 70.8% of workers being over the age of 45 years old. Workers belonging to the Indian ethnic group dominated the early morning (40.4%), night (46.5%), and mixed shift (38.8%) schedules. Night shift (28.3%) and early morning shift (25.5) workers were most likely to be from lower SES. Night shift workers had a higher prevalence of having diabetes (27.3%), CVD (14.1%), and being ever smokers (54.5%) compared to workers on other shift schedules. No significant differences were found between the groups for BMI, low SES, and hypertension.

### 3.3. Associations of Shift-Type, Cardiometabolic Risk Factors, and Cardiometabolic Disease

Workers on evening, night, and mixed schedules were more likely to be smokers compared to non-shift workers in the crude model (Table 2). Adjustments for various confounders did not affect the effect estimates, except for the night shift schedule, which attenuated and became non-significant. Night shift workers were also more likely to have hypertension compared to non-shift worker in the crude model, but this relationship attenuated after adjusting for confounders. No significant associations were found between shift type and BMI.

Similarly, night shift workers were more likely to have diabetes and CVD in the crude model (Table 2). However, after adjusting for sociodemographic factors (age, gender, ethnicity, and socioeconomic status) and work arrangement, the associations for both outcome measures became non-significant.

Further analyses on smokers were conducted by further stratifying smokers into past, current, and never smoker categories (Table 3). After adjusting for all covariates, it was found that evening shift workers were significantly more likely to be past smokers, night shift workers were significantly more likely to be current smokers, and mixed shift workers were equally likely to be past and current smokers.

### 3.4. Associations of Shift Duration (in Years) with Cardiometabolic Risk Factors, Diabetes, and Cardiovascular Disease

In the crude model, shift duration was significantly associated with smoking status, hypertension, and BMI (Table 4). Adjusting for sociodemographic factors of age, gender, ethnicity, and SES did not affect the associations between shift duration and smoking status. However, the association with BMI and hypertension was attenuated. Only the association between shift duration and smoking status remained significant in the fully adjusted model.

Shift duration was significantly associated with diabetes and CVE in the unadjusted model (Table 4). However, when fully adjusted for sociodemographic factors and work arrangement, the strength of these associations became attenuated, with no significant associations observed.

## 4. Discussion

In this study, we observed that, among shift workers, the prevalence of self-reported diabetes and CVD were significantly higher in night shift workers. However, these associations were attenuated after adjusting for ethnicity and low socioeconomic status. We also observed that evening night and mixed shift workers were more likely to be ever current smokers than non-shift workers, and that longer exposure to shift work (i.e., shift duration, in years) was associated with smoking, which remained significant after adjusting for potential confounders.

Even though shift work was not independently associated with cardiometabolic outcomes in adjusted analyses, shift types remained highly associated with having a history of smoking with smoking status (past or current). Previous studies have extensively shown that shift work is related to smoking behavior status [11,21,22,23,24] and individuals who had participated in shift work were found to have had an increased likelihood of consuming stimulants, including current tobacco use. Moreover, the association between shift work and smoking has been reported to be independent of other factors, such as SES [24].

In this study, only evening and mixed shift workers were more likely to be smokers in the final model. Evening and night shift workers were more likely to be associated with past and current smoking behaviors, respectively, while mixed shift workers were associated with both past and present smoking behaviors. The links between shift work and stimulant consumption (including cigarettes) have been well documented. In one study, individuals who had participated in shift work were found to have had an increased likelihood of consuming stimulants, including current tobacco users who had participated in shift work were found to have had an increased likelihood of consuming stimulants, including current smoking behavior [21]. A recent study investigating the lifestyle behaviors of night shift nurses found that, among these nurses, there was a 65% prevalence of nicotine dependence [22]. Smoking has also been shown to mediate the relationship between shift work and cardiometabolic health outcomes, such as obesity [11]. Furthermore, the likelihood of being an ever smoker increased by 2% with a longer duration spent doing shift work.

This finding is novel and adds to the literature, as very few studies have examined the effects of shift duration on unhealthy habits, such as smoking [25]. The differences between evening and night shift types and smoking categories could be due to night shift workers showing stronger tendencies towards substance reliance, possibly leading to addiction. It is possible that night shift workers, defined in this study as working between 9 p.m. and 6 a.m. (i.e., overnight), as well as mixed shift workers who would also have to conduct similarly defined night shifts, are at risk of circadian misalignment, which may increase the risk of substance abuse, as circadian mechanisms might be responsible for dopaminergic regulatory functions [26,27]. Furthermore, disruptions to the sleep cycle and circadian rhythm will lead to fatigue and, in turn, lower alertness and levels of concentration [28], hence night shift workers may resort to smoking to counter these effects [29]. In fact, one study has shown that it is almost impossible for smoking cessation programs to yield much success amongst night shift workers in particular [30]. Therefore, more studies are needed to uncover the long-term associations between night shift work and unhealthy habits and smoking behavior, and their consequences on cardiometabolic health.

For the association between shift work and CMD, the results from the crude model for both shift work type and shift duration are in line with findings from the literature. Night shift work has been shown to have an impact on cardiometabolic health. For example, one study showed that night shift work was linked to an increased risk of metabolic disorders [25]. There exists plenty of research indicating shift work has been associated with an increased risk of diabetes [31]. Similarly, longer durations of shift work have been shown to have strong links with the risk of diabetes [32]. Shift workers who were exposed to longer shift work duration (in years) and more frequent night shifts had a higher risk of developing CMD. In one study, it was found that the risk of cardiovascular events increased by 7.1% after every five years of exposure to shift work [27]. The mechanisms underlying the associations linking night shift work and CMDs, such as diabetes and cardiovascular diseases, are well known. Studies have shown that diabetes may be linked to reductions in melatonin levels [33], as well as circadian desynchronization by way of disrupted and/or low-quality sleep [34,35]. Additionally, night shift work, possibly due to fragmented sleep and/or disruptions to the circadian rhythm, has also been linked to inflammation and thickening of carotid arteries, which is a risk factor for many cardiovascular diseases [36,37,38]. However, the association between shift work and diabetes became non-significant after adjusting for ethnicity and SES. We propose that individuals of lower SES may be more prone to engage in unhealthy or risky health behaviors [39]. Additionally, at least one study has shown that significant variations in blood pressure and total sleep time exist amongst shift workers based on ethnicity, which may lead to negative health outcomes attributed to shift work [20]. These may explain the results for the association between shift work and CMD in the present study.

Our study did not find a significant association between shift type and shift duration on hypertension, or BMI levels after adjusting for covariates. Many studies have shown that, compared to non-shift workers, shift workers are significantly more overweight, independent of other effects, such as dietary intake [21,23]. The null findings from this study could be attributed to the fact that the participants recruited were from a generally healthier population [14]. Furthermore, other studies have also reported that shift workers who were non-smokers and had prolonged exposure to shift work may be more overweight than shift workers who were smokers [11]. Despite this, there is little consensus on the directionality of the association between smoking and obesity. However, some studies have shown that smoking is positively associated with increased obesity after accounting for endogenous factors [40]. Therefore, future research on shift work, obesity, and smoking habits should also account for endogenous factors, such as individual dietary habits. Additionally, at least one study has shown that increased BMI and smoking habits may increase the risk of COVID-19 infection among night shift workers [41].

The results for hypertension were not surprising. Most literature findings have yielded mixed associations between shift work and hypertension [42,43,44]. Although studies have shown links between shift workers and hypertension, this link may be moderated by sleep duration, which was not factored into the analyses of the present study [44]. Further studies with more robust methodologies are required to assess the causal links between shift work and hypertension.

The findings from this study should be interpreted while keeping in mind some limitations. Firstly, this study retrospectively analyzed data from an already established cohort, which meant little control over the data’s collection. However, all interviewers had been trained for accurate and consistent data collection [14]. Second, the method in which shift work data was obtained could be prone to bias. In the SP2PAQ, participants were asked to state their shift work schedules for early morning, evening, and night. While this provided granularity to the data, this could be subjected to recall bias, causing some shift types to be recalled more frequently than others. There was also no question to confirm that such work was perceived by the participants as shift work. As such, the appropriate comparison group was manually derived. While this allows for a standardized assessment of shift work in this analysis, there may be some inaccuracies in comparison to participants’ perceptions of shift work. In addition, there was also a variation with regard to the demarcation of each shift work category in terms of time (e.g., 4 p.m.–9 p.m. for the evening shift vs. 9 p.m.–6 a.m. for the night shift). These variations in shift work categories is a limitation, as it could have implications on circadian mechanisms that affect cardiometabolic health outcomes [12]. Third, while the original study population was large, many participants (approximately 60%) were removed due to missing shift work data, which may have introduced selection bias, due to differences between participants who were excluded and those included in the study (Supplementary Table S1). Fourth, this study uses a cross-sectional design, hence the associations are not causal [45]. Finally, we lacked information on the number of cigarettes smoked, which may have led to the loss of information and residual confounding.

Despite these limitations, this study also had several strengths. The main strength of this study is that it utilized a multi-ethnic study population, and is the first study to explore the possible relationships between shift work and cardiometabolic health outcomes in a Singapore-based cohort. Utilizing a multi-ethnic cohort allowed this study to move beyond specific ethnic groups so as to have good representation and comparability across all ethnicities. The cross-sectional design utilized in this study allowed for quick and effective measurements of the prevalence and measurements of association for multiple variables at once [45]. Additionally, as the MEC revisit of phase 1 provided a comprehensive list of variables, data for various covariates, such as age, gender, ethnicity, and SES, were available. Finally, the data from this study were obtained from participants representing the general population; therefore, the findings from this study can be generalized to a larger population.

In Singapore, the attributable population risk of CMD caused by cigarette smoking has been shown to be between 4–12% [46]. Therefore, there is a pressing need to address the issue of smoking among shift workers. Workplace-based smoking interventions targeted at individuals, such as providing counseling, self-help materials, environmental cues, social support, etc., have been successful in helping individuals toward smoking cessation [47]. Future studies in Singapore should investigate the in-depth nature of shift work and its relation to health-seeking behaviors and behaviors that lead to adverse health outcomes. Future studies should also test the efficacy of various interventions in workplaces requiring employers to work in different shift schedules (i.e., hospitals, transport companies, etc.).

## 5. Conclusions

Evening and mixed shift schedules, as well as shift duration, were significantly associated with smoking status. The findings from this study should help inform guidelines for companies that employ shift workers. Furthermore, it should encourage an increase in health promotion interventions (e.g., smoking cessation drives) to lead to a healthier workforce. Future studies in Singapore should investigate the in-depth nature of shift work and its relation to health-seeking behaviors, and behaviors that lead to adverse health outcomes.

## Figures and Tables

**Table 1:** Demographic characteristics of shift workers stratified according to their reported shift schedule types.

Variables	Non-Shift	Early Morning	Evening	Night	Mixed	*p*-Value *
*n* (%) **	1831 (74)	47 (7.4)	268 (42.0)	99 (15.5)	224 (35.1)	
Shift duration (years), M (SD)	-	8.61 (9.5)	7.41 (8.4)	9.76 (10.3)	9.69 (9.5)	**<0.027**
Gender						
Male, *n* (%)	833 (45.5)	17 (36.2)	129 (48.1)	67 (67.7)	152 (67.9)	**<0.001**
Age Range						
<35, *n* (%)	318 (17.4)	7 (14.9)	57 (21.3)	15 (15.2)	63 (28.1)	**<0.01**
35–44, *n* (%)	439 (24.0)	7 (14.9)	59 (22.0)	14 (14.1)	56 (25.0)	
45–54, *n* (%)	565 (30.9)	18 (38.3)	81 (30.2)	35 (35.4)	59 (26.3)	
≥55, *n* (%)	509 (27.8)	15 (31.9)	71 (26.5)	35 (35.4)	46 (20.5)	
Ethnicity						
Chinese, *n* (%)	729 (39.8)	13 (27.7)	115 (42.9)	13 (13.1)	52 (23.2)	**<0.001**
Malay, *n* (%)	337 (18.4)	8 (17.0)	41 (15.3)	25 (25.3)	58 (25.9)	
Indian, *n* (%)	598 (32.7)	19 (40.4)	84 (31.3)	46 (46.5)	87 (38.8)	
Other, *n* (%)	167 (9.1)	7 (14.9)	28 (10.4)	15 (15.2)	27 (12.1)	
Low SES						
Yes, *n* (%)	303 (16.5)	12 (25.5)	47 (17.5)	28 (28.3)	40 (17.9)	**0.02**
Work Arrangement						
Part Time, *n* (%)	346 (18.9)	7 (14.9)	56 (20.9)	14 (14.1)	19 (8.5)	**0.001**
Smoking Status						
Ever smoker, *n* (%)	468 (25.6)	14 (29.8)	92 (34.3)	45 (54.5)	106 (47.5)	**<0.001**
Hypertension						
Yes, *n* (%)	384 (21.0)	11 (23.4)	49 (18.3)	30 (30.3)	42 (18.8)	0.13
BMI						
≥25 kg/m^2^, *n* (%)	773 (51.9)	23 (60.5)	116 (43.3)	50 (63.3)	91 (55.8)	0.25
Diabetes						
Yes, *n* (%)	251 (13.7)	8 (17.0)	39 (14.6)	27 (27.3)	30 (13.4)	**<0.01**
CVE						
Yes, *n* (%)	107 (5.8)	1 (2.1)	17 (6.3)	14 (14.1)	10 (4.5)	**<0.01**

BMI = body mass index, M = mean, *n* = number of shift workers, SD = standard deviation, CVE = cardiovascular event, SES = socioeconomic status. * Bolded values indicate that significant differences were found in ANOVA or Pearson’s chi-square tests. ** Percentage (%) of non-shift workers was calculated by using the total number of included participants as the denominator (*n* = 2469), while the respective shift-work categories was calculated by using the total number of shift-workers (*n* = 638) as the denominator (see Supplementary Figure S1).

**Table 2:** Binary logistic regression of associations between shift schedule type and CMD risk factors and disease outcomes.

Model	Smoking Status	Hypertension	BMI	Diabetes	Cardiovascular Event
*n*	2467	2465	1986	2469	2469
	OR (95%CI)	OR (95%CI)	OR (95%CI)	OR (95%CI)	OR (95%CI)
Crude					
Non-Shift	1	1	1	1	1
Morning	1.24 (0.66–2.33)	1.15 (0.58–2.28)	1.42 (0.73–2.74)	1.29 (0.60–2.80)	0.35 (0.05–2.56)
Evening	1.52 (1.16–2.00) **	0.85 (0.61–1.18)	1.05 (0.79–1.40)	1.07 (0.74–1.54)	1.09 (0.64–1.85)
Night	2.43 (1.61–3.65) ***	1.64 (1.05–2.55) *	1.60 (1.00–2.55)	2.36 (1.49–3.75) ***	2.65 (1.46–4.83) **
Mixed	2.64 (1.99–3.50) ***	0.87 (0.61–1.24)	1.17 (0.84–1.62)	0.97 (0.65–1.46)	0.75 (0.39–1.46)
Model 1 ^a^					
Non-Shift	1	1	1	1	1
Morning	1.59 (0.78–3.23)	1.02 (0.49–2.12)	1.32 (0.67–2.63)	1.01 (0.45–2.29)	0.30 (0.04–2.29)
Evening	1.59 (1.16–2.18) **	0.86 (0.61–1.23)	1.15 (0.86–1.56)	1.15 (0.78–1.71)	1.08 (0.63–1.88)
Night	1.55 (0.97–2.48)	1.16 (0.71–1.89)	1.20 (0.74–1.95)	1.58 (0.96–2.62)	1.78 (0.92–3.43)
Mixed	1.69 (1.22–2.33) **	0.94 (0.64–1.39)	1.04 (0.74–1.460)	1.00 (0.65–1.56)	0.81 (0.41–1.63)
Model 2 ^b^					
Non-Shift	1	1	1	1	1
Morning	1.57 (0.77–3.20)	1.02 (0.49–2.12)	1.23 (0.65–2.58)	1.00 (0.44–2.27)	0.30 (0.41–2.29)
Evening	1.60 (1.17–2.20) **	0.86 (0.61–1.23)	1.16 (0.86–1.57)	1.16 (0.78–1.72)	1.08 (0.63–1.88)
Night	1.55 (0.97–2.48)	1.16 (0.71–1.89)	1.19 (0.73–1.94)	1.58 (0.95–2.62)	1.78 (0.92–3.43)
Mixed	1.68 (1.22–2.32) **	0.94 (0.64–1.39)	1.03 (0.73–1.45)	0.99 (0.64–1.53)	0.81 (0.41–1.63)

^a^ Adjusted for age, gender, ethnicity, and SES (model1). ^b^ Model 1 + work arrangement (model 2). * *p* < 0.05. ** *p* < 0.01. *** *p* < 0.001.

**Table 3:** Multinomial logistic regression of associations between shift schedule type and smoking status stratified according to past and current smokers.

Model	Past Smoker (*n* = 246)	Current Smoker (*n* = 479)
	OR (95%CI)	OR (95%CI)
Crude		
Non-Shift	1	1
Morning	1.01 (0.35–2.88)	1.36 (0.66–2.79)
Evening	1.75 (1.18–2.58) **	1.40 (0.61–1.18) *
Night	1.08 (0.48–2.41)	3.15 (2.04–4.86) ***
Mixed	2.41 (1.59–3.65) ***	2.76 (2.01–3.79) ***
Model 1 ^a^		
Non-Shift	1	1
Morning	1.31 (0.43–4.00)	1.72 (0.75–3.93)
Evening	1.80 (1.19–2.71) **	1.41 (0.98–2.03) *
Night	0.74 (0.32–1.70)	2.08 (1.27–3.42) **
Mixed	1.73 (1.12–2.68) *	1.76 (1.23–2.51) **
Model 2 ^b^		
Non-Shift	1	1
Morning	1.32 (0.43–4.01)	1.71 (0.75–3.90)
Evening	1.80 (1.19–2.71) **	1.42 (0.99–2.05)
Night	0.74 (0.32–1.70)	2.10 (1.26–3.41) **
Mixed	1.72 (1.11–2.67) *	1.74 (1.22–2.48) **

^a^ Adjusted for age, gender, ethnicity, and SES (model1). ^b^ Model 1 + work arrangement (model 2). * *p* < 0.05. ** *p* < 0.01. *** *p* < 0.001.

**Table 4:** Binary logistic regression of associations between shift duration (years) and CMD risk factors and disease outcomes.

Model	Smoking Status	Hypertension	BMI	Diabetes	Cardiovascular Event
*n*	2410	2408	1941	2412	2412
	OR (95%CI)	OR (95%CI)	OR (95%CI)	OR (95%CI)	OR (95%CI)
Crude	1.02 (1.01–1.04) ***	1.02 (1.00–1.03) *	1.02 (1.00–1.04) *	1.03 (1.02–1.05) **	1.02 (1.00–1.05) *
Model1 ^a^	1.02 (1.00–1.04) *	1.00 (0.98–1.01)	1.01 (0.99–1.03)	1.01 (0.99–1.03)	1.00 (0.98–1.03)
Model 2 ^b^	1.02 (1.00–1.03) *	1.00 (0.98–1.01)	1.01 (0.99–1.03)	1.01 (0.99–1.03)	1.00 (0.98–1.03)

^a^ Adjusted for age, gender, ethnicity, and SES (model1). ^b^ Model 1 + work arrangement (model 2). * *p* < 0.05. ** *p* < 0.01. *** *p* < 0.001.

## Data Availability

Data described in the manuscript, code book, and analytic code will be made available upon request pending application and approval from the corresponding author.

## Supplementary Materials

The following are available online at https://www.mdpi.com/article/10.3390/ijerph20032047/s1, Figure S1: Flowchart of MEC revisit of phase 1 participants included in the analyses of this study; Table S1: title Differences in the characteristic between included and excluded participants.
