# Supplementary material for: Association between Shift Work Schedules and Cardiovascular Events in a Multi-Ethnic Cohort
Source: Int J Environ Res Public Health. 2023 Jan 22;20(3):2047. doi: 10.3390/ijerph20032047 (PMC9916120; doi:10.3390/ijerph20032047)
Supplement: Supplementary file 1 [file ijerph-20-02047-s001.zip › ijerph-2055004-supplementary.pdf]

Supplementary Figure S1. Flowchart of MEC revisit of phase 1 participants included in the analyses of this study

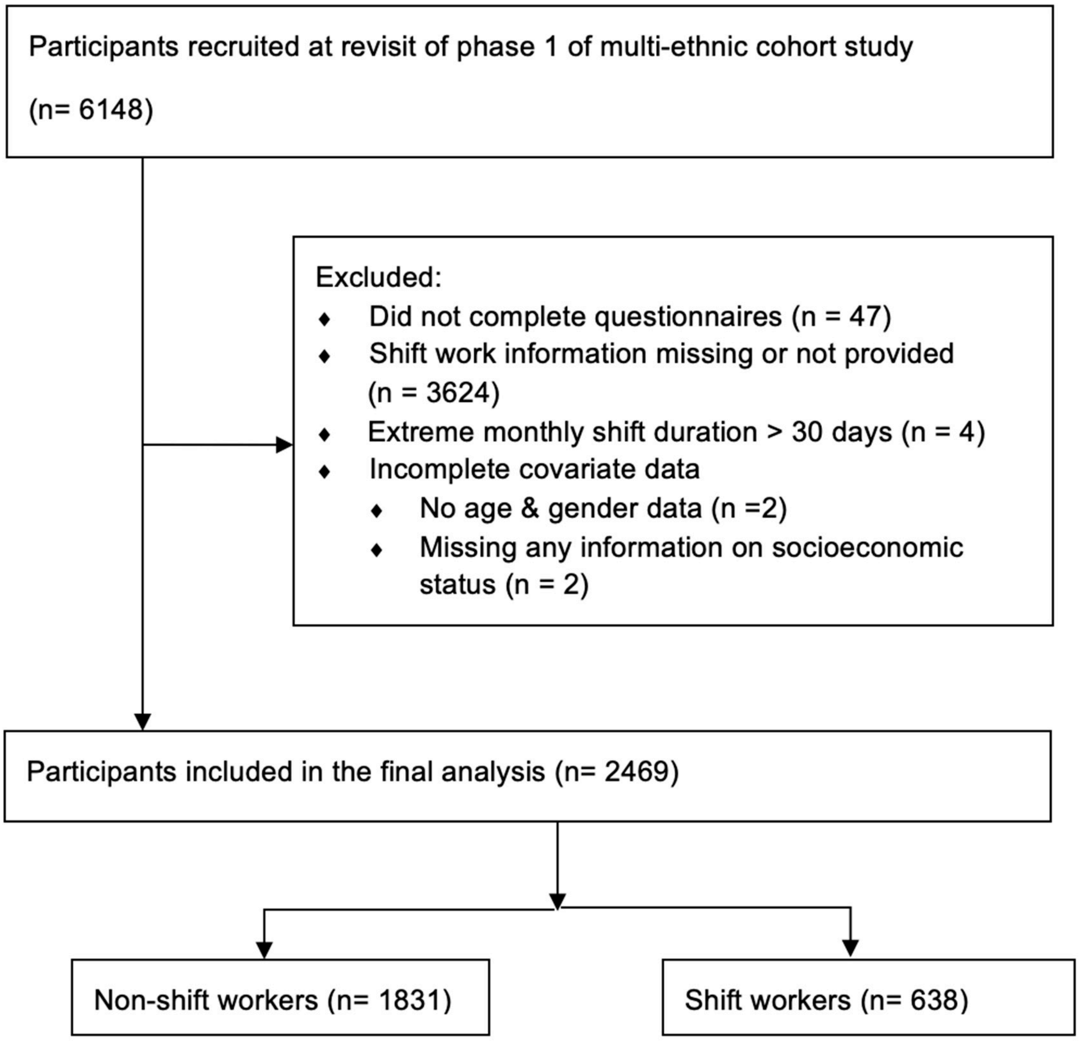

Supplementary Table S1. Differences in the characteristic between included and excluded participants

Variables	Total	Excluded	Included	p-value*
**n**	**6101**	**3632**	**2469**	
**Age (years), M (SD)**	52.1 (12.3)	50.4 (13.3)	54.8 (10.0)	**< 0.001**
**Gender**				
Male, n (%)	2579 (42.3)	1481 (40.8)	1198 (54.8)	**< 0.001**
**Ethnicity**				
Chinese n (%)	2827 (46.3)	1128 (31.1)	1699 (68.8)	**< 0.001**
Malay n (%)	1073 (17.6)	768 (21.1)	305 (12.4)	
Indian n (%)	1650 (27.0)	1302 (35.8)	348 (14.1)	
Other n (%)	551 (9.0)	434 (11.9)	117 (4.7)	
**Low SES**				
Yes, n (%)	1194 (19.6)	802 (22.1)	392 (15.9)	**< 0.01**

Table footnotes:

*Bolded values indicate that significant differences were found in Student t-test or Pearson’s chi-square tests
